# Serum levels of soluble programmed death-ligand 1 (sPD-L1) in patients with primary central nervous system diffuse large B-cell lymphoma

**DOI:** 10.1186/s12885-020-6612-2

**Published:** 2020-02-13

**Authors:** Inju Cho, Hansang Lee, Sang Eun Yoon, Kyung Ju Ryu, Young Hyeh Ko, Won Seog Kim, Seok Jin Kim

**Affiliations:** 1 Department of Pathology, Yeouido St. Mary’s Hospital, The Catholic University of Korea, Seoul, South Korea; 2 Department of Internal Medicine, Dankook University College of Medicine, Cheonan, South Korea; 3 Division of Hematology and Oncology, Department of Medicine, Samsung Medical Center, Sungkyunkwan University School of Medicine, 81 Irwon-ro, Gangnam-gu, Seoul, 06351 South Korea; 4 Department of Health Sciences and Technology, Samsung Advanced Institute for Health Sciences and Technology, Sungkyunkwan University, Seoul, South Korea; 5 Department of Pathology and Translational Genomics, Samsung Medical Center, Sungkyunkwan University College of Medicine, Seoul, South Korea

**Keywords:** Soluble PD-L1, PD-1, Primary central nervous system lymphoma

## Abstract

**Background:**

The interaction of programmed death-1 protein (PD-1) and programmed death-1 ligand (PD-L1) produces immunosuppressive activity, protecting tumor cells from anti-tumor immunity and possibly releasing soluble PD-L1 (sPD-L1) from PD-L1 expressing tumor cells. Therefore, we measured serum levels of sPD-L1 in patients with primary central nervous system lymphoma (PCNSL) and explored its clinical implications.

**Methods:**

Sixty-eight patients with newly diagnosed PCNSL had diffuse large B-cell lymphoma and were treated with high-dose methotrexate-containing chemotherapy. The measurement of sPD-L1 and cytokines was performed using serum samples archived at diagnosis, and the tissue expression of PD-L1 was also analyzed from archived paraffin-embedded tissue blocks. Disease relapse, progression-free survival (PFS), and overall survival (OS) were analyzed according to the extent of sPD-L1 in serum and PD-L1 in tissue.

**Results:**

The median level of serum sPD-L1 (0.429 ng/mL) was higher than in healthy control patients (0.364 ng/mL). The occurrence of relapse was more frequent in the high sPD-L1 (78%) than the low sPD-L1 group (50%), though the groups did not have different clinical or pathological characteristics at diagnosis. As a result, the OS and PFS for the high sPD-L1 group were significantly lower than those in the low group. PD-L1-positive tumor cells were found in 35 patients (67%), and the extent of PD-L1-postive tumor cells was positively associated with serum sPD-L1 levels (*r* = 0.299, *P* = 0.031). Among the 34 cytokines analyzed, only the serum level of IL-7 correlated with the serum level of sPD-L1 (r = 0.521, *P* < 0.001).

**Conclusions:**

Serum levels of sPD-L1 could reflect the expression of PD-L1 in PCNSL tumor cells and predict patient survival outcomes. Therefore, sPD-L1 in serum could be a feasible biomarker for determining a risk-adapted treatment strategy for PCNSL patients.

**Trial registration:**

The study population was patients who were diagnosed with PCNSL between January 2009 and February 2017 and registered for our prospective cohort studies after providing written informed consent (ClinicalTrials.gov: NCT00822731 [date of registration - January 14, 2009] and NCT01877109 [date of registration - June 13, 2013]).

## Background

Primary central nervous system lymphoma (PCNSL) is a rare but aggressive non-Hodgkin lymphoma (NHL) that is confined to the brain, spinal cord, leptomeninges, and eyes [1]. Most cases of PCNSL have diffuse large B-cell lymphoma (DLBCL) histopathology, but PCNSL accounts for less than 3% of all primary tumors of the CNS and 1 to 2% of all NHLs [2–4]. Currently, high-dose methotrexate (HD-MTX) is the backbone of the multi-agent chemotherapies used, and HD-MTX-containing chemotherapy with or without whole brain radiotherapy (WBRT) has produced response rates of 70 to 90% in patients newly diagnosed with PCNSL [5–9]. However, almost 50% of patients relapse within the first two years after diagnosis, and one-third become refractory to conventional chemotherapy [10–12]. Salvage treatment options for relapsed/refractory PCNSL include high-dose chemotherapy followed by autologous stem cell transplantation (ASCT) or WBRT [13–15]. However, responses to those treatments are usually not durable, and ASCT cannot be used for frail elderly patients. Given the high probability of treatment failure and limited treatment options for frail elderly patients, the development of more effective salvage treatment with less toxicity has been an unmet need for PCNSL.

PD-1 is an inhibitory receptor expressed on activated T cells, and its interaction with PD-L1 suppresses T-cell mediated immune response allowing tumor cells to escape from anti-tumor immunity [16]. As immune checkpoint inhibitors blocking PD-1 have shown their efficacy in relapsed or refractory lymphoma patients, the role of PD1 inhibitor as a salvage treatment also has been emerging in patient with PCNSL. Indeed, nivolumab showed single-agent activity in relapsed and refractory PCNSL patients, and frequent copy-number alterations in *9p24.1/PD-L1/PD-L2* of tumor cells leading to the expression of PD-L1 and PD-L2 were found in PCNSL [17, 18]. However, the assessment of PD-L1 and L2 in tumor tissue is not always possible in patients with PCNSL due to the risk of post-biopsy complications. The soluble programmed death-ligand 1 (sPD-L1) is secreted from PD-L1 positive cells, and can easily be measured using an enzyme-linked immunosorbent assay (ELISA) [19]. Thus, the measurement of sPD-L1 might become an indirect marker reflecting the expression of PD-L1 in tumor tissue. Indeed, elevated levels of sPD-L1 were reported to affect overall survival in DLBCL patients in a previous French multi-center trial [20]. In this study, we measured the level of sPD-L1 in patients with PCNSL and analyzed its clinical relevance as a prognostic marker, as well as its correlation with PD-L1 expression in tumor cells.

## Methods

### Patients

The study population was patients who were diagnosed with PCNSL between January 2009 and February 2017 and registered for our prospective cohort studies after providing written informed consent (NCT00822731 and NCT01877109). In our prospective cohort studies, we collected serum samples and the pre-treatment characteristics of patients at diagnosis. Treatment and outcome-related data, including treatment regimens, tumor response, date of progression, and date of death, were regularly updated. These cohort studies were approved by the Institutional Review Board of Samsung Medical Center, and all investigations were conducted according to the principles expressed in the Declaration of Helsinki and its contemporary amendments. Because patients with all subtypes of lymphoma were enrolled, the evaluations for work-up and treatments were performed according to our clinical practice for each subtype. For patients with PCNSL, the initial evaluation was done according to the International Primary CNS Lymphoma Collaborative Group recommendations [21]. Cerebrospinal fluid (CSF) analyses and ophthalmic examinations were also performed in most patients to test for leptomeningeal and ocular invasion. As the primary treatment for newly diagnosed PCNSL, HD-MTX-containing chemotherapy with or without WBRT was used. Response was assessed according to the response criteria for PCNSL recommended by the International Primary CNS Lymphoma Collaborative Group [21]: complete response (CR) was defined as no contrast enhancement in brain magnetic resolution imaging (MRI) and negative findings in ocular and CSF examinations; partial response (PR) was defined as at least a 50% decrease in the enhancing tumor lesion; progressive disease (PD) was defined as at least a 25% increase in the lesion or any new lesion in the CNS or systemic sites; and stable disease (SD) was defined as less than a PR but not PD. Response evaluation was performed after the completion of primary treatment chemotherapy, and surveillance brain MRI was done to monitor the occurrence of disease relapse.

### Study design

We retrospectively analyzed 68 patients who had archived serum samples available for measurement of sPD-L1 among patients enrolled in the aforementioned cohort studies, after excluding patients with secondary CNS involvement in systemic DLBCL. Using serum samples and ELISA, we first measured the sPD-L1 levels and correlated them with the clinical and pathological characteristics of the patients at diagnosis. Then, response to first-line therapy and the survival outcomes of patients were compared according to the level of sPD-L1. Second, we analyzed the expression of PD-L1 in tumor cells and non-tumor cells in 52 patients whose paraffin-embedded tissue blocks were available for immunohistochemistry analyses. Third, we measured serum cytokines using multiplex ELISA to explore additional biomarkers that might predict the outcomes of PCNSL patients and influence the level of sPD-L1 or the tissue expression of PD-L1. To confirm the DLBCL histology of our patients with PCNSL, two pathologists (I.C and Y.K) reviewed patients’ histopathology slides using the 2017 World Health Organization classification [3]. Relapsed disease was defined as disease recurrence in patients who had no evidence of disease after cessation of therapy, and PD was defined as SD or PD during the primary treatment. Multiple diseases were defined as more than one lesion found in a radiologic evaluation, and deep regions of the brain were defined as the basal ganglia, brainstem, periventricular regions, and cerebellum. We updated the survival status in March 2019 for the survival analysis, and this study was approved by the Institutional Review Board of Samsung Medical Center (IRB No. 2019–05-054).

### Measurement of serum sPD-L1

Serum samples were collected at diagnosis and stored at − 80 °C until analysis. Serum aliquots had not been previously thawed before use in our multiplex chemokine assay. The level of sPD-L1 was measured using ELISA kits (PDCD1LG1 ELISA kit, USCN Life Science, Wuhan, China) according to the manufacturers’ instructions. Briefly, the microplate provided in the kit was pre-coated with an antibody specific to PDCD1LG1. Standards or samples were then added to the microplate wells with a biotin-conjugated antibody specific to PDCD1LG1. Next, avidin conjugated to horseradish peroxidase was added to each microplate well and incubated. After the enzyme-substrate reaction, the color change was measured spectrophotometrically at a wavelength of 450 nm. To estimate the reference ranges of sPD-L1, we measured the levels of serum sPD-L1 in 12 normal individuals (6 males and 6 females, median age 51 (range 24–78). They voluntarily donated residual serum samples that were left after blood tests during their regular health check-up. The sPD-L1 values in the blood serum specimens of healthy controls were determined by the same method. The measurement of each sample was done in duplicate.

### Immunohistochemistry for tissue PD-L1 expression

Immunohistochemistry was performed on paraffin tissue sections (4-μm thick), and the PD-L1 antibody (Spring Bioscience, CA, USA; clone SP142, M4421, rabbit anti-human PD-L1/CD274, monoclonal antibody, 1:25 dilution) was used to assess the expression of PD-L1. The antibody was incubated for 120 min at 37 °C using the Ventana BenchMark XT platform after antigen retrieval for 92 min with CC1 buffer. Signal visualization was done using the OptiView DAB immunohistochemistry detection kit (Ventana, Tucson, Azusa) and OptiView Amplification kit (Ventana, Tucson, Azusa). Tonsil squamous epithelium was used as a PD-L1 immunohistochemistry positive control [22]. The slides were semi-quantitatively analyzed by two pathologists (I. C and Y. K). The extent of PD-L1 expression in tumor cells was defined as the proportion of tumor cells showing PD-L1 expression with any intensity in the tumor area [23]. Macrophages and lymphocytes infiltrating the tumor area were considered non-tumor immune cells, and the proportion of PD-L1 expression in them was assessed in the same manner as in the tumor cells. Tumor cells were discriminated from tumor infiltrating lymphocytes using morphology because the tumor cells had unequivocal morphologic characteristics that allowed discernment. The assessment of PD-L1 expression in tumor infiltrating macrophages was done by measuring PD-L1 expression in CD68-positively stained macrophages. PD-L1-positive tumor cells were defined as those positively stained for PD-L1 with a distinct membranous, cytoplasmic, or punctate/granular pattern of any intensity based on previously published descriptions [23, 24]. The following additional antibodies were used to assess CD68 expression and identify the cell of origin: CD68 (Leica Biosystem, Newcastle, NCL-L-CD68, mouse monoclonal, 1:50 dilution), CD10 (Novocastra, Newcastle, NCL-L-CD10–270, mouse monoclonal, 1:100 dilution), BCL6 (Novocastra, Newcastle, NCL-L-Bcl-6-564, mouse monoclonal, 1:80 dilution), and MUM1 (Dako, CA, M7259, mouse monoclonal, 1:100 dilution). To assess the positivity of Epstein-Barr virus (EBV) in tumor tissue, EBV-encoded RNA (EBER) in situ hybridization (ISH) was also performed because EBV-positivity could be associated with PD-L1 expression. EBER was detected using ISH and an EBV ISH kit (Leica Microsystems, Bannockburn, IL, USA). We used EBV-negative lymphoid tissues and the hybridization mixture without EBV oligonucleotides as negative controls.

### Multiplex cytokine assay

We measured eotaxin-1, GROα, interferon (IFN)-α, IFN-γ, IL-1α, IL-1β, IL-1RA, IL-2, IL-4, IL-5, IL-6, IL-7, IL-8, IL-9, IL-10, IL-12p70, IL-13, IL-15, IL-17α, IL-18, IL-21, IL-22, IL-23, IL-27, IL-31, interferon γ-induced protein (IP-10), monocyte chemoattractant protein 1 (MCP-1), macrophage inflammatory protein-1α (MIP-1α), MIP-1β, regulated on activation T cell expressed and secreted (RANTES), stromal cell-derived factor 1α (SDF1α), tumor necrosis factor (TNF)-α, and TNF-β levels in duplicate with a ProcartaPlex™ multiplex immunoassay kit (Invitrogen, Camarillo, CA, USA) and the Bio-Plex Cytokine Assay System (Bio-Rad Laboratories, Hercules, CA, USA) according to the manufacturer’s instructions.

### Statistical analysis

In the survival analysis, overall survival (OS) was designated as the time from the date of diagnosis to the date of death or last follow-up and progression-free survival (PFS) was designated as the time from the date of diagnosis to the date of progression or relapse, death, or last follow-up. The optimal cutoff value for the survival analysis was determined using the receiver-operating characteristic (ROC) curve method. Kaplan-Meier survival graphs and log-rank tests were used for the univariate survival analyses, and Cox proportional regression models were used for the multivariate analyses. A bi-variate analysis of sPD-L1 and tissue expression of PD-L1 was done to analyze their correlation. The chi-square test or Fisher’s exact test were used to analyze their associations with clinical and pathological characteristics. In all comparisons, *P*-values less than 0.05 were considered statistically significant, and all P-values correspond to two-tailed significance tests. Statistical analyses were carried out using SPSS software version 21.0 (IBM, Armonk, New York, USA).

## Results

### Characteristics and treatment outcomes of patients

The median age of the 68 patients was 55 years (range: 20–77 years), and 40% of the patients were older than 60 years at diagnosis (Table 1). Female patients were predominant, and most patients had good performance status (Eastern Cooperative Oncology Group grade 0–1). There were no patients with known immunosuppression such as the history of post-organ transplantation state or human immunodeficiency virus infection or immunosuppressive medications. The majority of patients had multiple lesions in the CNS, including 8 cases with combined leptomeningeal and parenchymal involvement, and twenty-seven patients (35%) with a tumor in the deep region of the brain (Table 1). Histologically, the activated B-cell (ABC) type (74%) was dominant over the germinal center type (19%, Table 1). All patients received HD-MTX-containing chemotherapy as follows: MTX 3.5 g/m^2^ on day 1, vincristine 1.4 g/m^2^ on day 1, and procarbazine 100 mg/m^2^ on day 1–7 every two weeks (total 5 cycles). Among the 68 patients, 90% completed the planned cycle of HD-MTX chemotherapy (5 cycles in 10 weeks); 7 patients failed to complete the planned treatment due to lack of response or intolerance to treatment (Table 2). Intrathecal chemotherapy and WBRT were performed as adjuvant treatments at the physicians’ discretion. Although 97% of patients responded to the primary treatment, disease relapse occurred in 57% of patients, and ASCT was done in 12 patients after salvage therapy. At the time of analysis, 39 patients were alive, and 29 patients had died (Table 2).

**Table 1:** Patient characteristics

	All patients (*n* = 68)		Low sPD-L1 (*n* = 36)		High sPD-L1 (*n* = 32)		
	*n*	(%)	*n*	(%)	*n*	(%)	*P*
Age (years)							
≤ 60	41	(60)	22	(61)	19	(59)	0.884
> 60	27	(40)	14	(39)	13	(41)	
Sex							
Male	27	(40)	13	(36)	14	(44)	0.622
Female	41	(60)	23	(64)	18	(56)	
ECOG PS							
0–1	56	(82)	33	(92)	23	(72)	0.054
≥ 2	12	(18)	3	(8)	9	(28)	
Serum Lactate dehydrogenase							
Normal	54	(79)	29	(81)	25	(78)	> 0.999
Elevated	14	(21)	7	(19)	7	(22)	
Number of lesions							
Single	11	(16)	7	(19)	4	(12)	0.521
Multiple	57	(84)	29	(81)	28	(88)	
Brain deep region involvement							
Absence	44	(65)	23	(64)	21	(66)	> 0.999
Presence	24	(35)	13	(36)	11	(34)	
Cell of origin subtype							
GCB	13	(19)	8	(22)	5	(16)	0.719
ABC	50	(74)	25	(69)	25	(78)	
Not evaluated	5	(7)	3	(8)	2	(6)	
Ki67							
< 90	23	(34)	11	(31)	12	(38)	0.769
≥ 90	42	(62)	23	(64)	19	(59)	
Not evaluated	3	(4)	2	(6)	1	(3)

**Table 2:** Treatment and patient outcomes

	All patients (*n* = 68)		Low sPD-L1 (*n* = 36)		High sPD-L1 (*n* = 32)		
	*n*	(%)	*n*	(%)	*n*	(%)	*P*
HD-MTX chemotherapy							
Completed	61	(90)	32	(89)	29	(91)	> 0.999
Failed to complete	7	(10)	4	(11)	3	(9)	
Response to HD-MTX treatment							
CR/PR	54/12	(97)	30/6	(100)	24/6	(94)	0.486
SD/PD	1/1	(3)	0/0	(0)	1/1	(6)	
Intrathecal chemotherapy							
Combined	26	(38)	10	(28)	16	(50)	0.081
Not combined	42	(62)	26	(72)	16	(50)	
Whole brain radiotherapy							
Done	45	(66)	24	(67)	21	(67)	> 0.999
Not done	23	(34)	12	(33)	11	(34)	
Relapse after primary treatment							
No relapse	29	(43)	18	(50)	11	(34)	0.226
Relapse	39	(57)	18	(50)	21	(66)	
Autologous stem cell transplantation							
Done	12	(18)	7	(19)	5	(16)	0.758
Not done	56	(82)	29	(81)	27	(84)	
Relapse or progression							
None	25	(37)	18	(50)	7	(22)	0.023
Occurred	43	(63)	18	(50)	25	(78)	
Survival status							
Alive	39	(57)	27	(75)	12	(37)	0.003
Dead	29	(43)	9	(25)	20	(63)	

### Serum level of soluble PD-L1

**Fig. 1:**
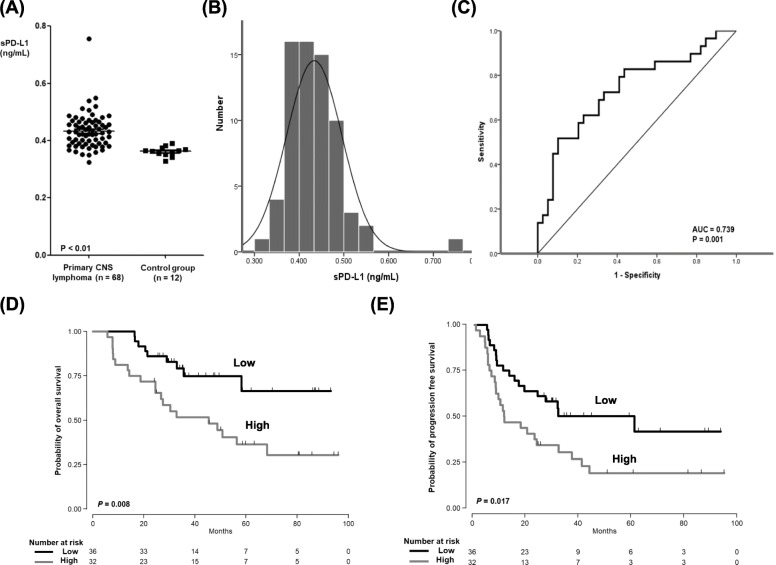
**a** Comparison of serum sPD-L1 between patients with PCNSL (*n* = 68) and the healthy control group (*P* < 0.01). **b** The distribution of serum sPD-L1 in 68 patients. **c** The ROC curve of sPD-L1 for overall survival. **d**, **e** The overall survival and progression-free survival in the high sPD-L1 group were lower than those in the low sPD-L1 group

The median level of patients’ serum sPD-L1 (*n* = 68) was 0.429 ng/mL (range: 0.324–0.757 ng/mL), which was significantly higher than in the healthy control group (0.364 ng/mL; range: 0.329–0.390 ng/mL, *P* < 0.01, Fig. 1a). The distribution of serum sPD-L1 among the 68 patients followed the normal distribution (Fig. 1b). The optimal cutoff for predicting OS was 0.432 ng/mL, and the area under the curve of sPD-L1 for OS was 0.739 (Fig. 1c). According to this cutoff value, we dichotomized patients into low and high groups (< 0.432 ng/mL versus ≥0.432 ng/mL). The OS and PFS of the high group were significantly lower than those of the low group (Fig. 1d, e). When we compared the low and high group patients’ pre-treatment characteristics at diagnosis, we found no significant differences between them (Table 1). In other words, unfavorable parameters such as multiple lesions, deep brain involvement, ABC type, and high Ki67 were not associated with the high sPD-L1 group. All patients received the same treatment: HD-MTX combination chemotherapy. Furthermore, their initial response to that therapy did not differ between groups either. However, relapse and progression were more frequent in the high sPD-L1 group (78%, 25/32) than in the low sPD-L1 (50%, 18/36) group (*P* = 0.023, Table 2). As a result, the number of deaths in the high group (*n* = 20) was significantly higher than in the low group (*n* = 9, Table 2).

### Tissue expression of PD-L1

**Fig. 2:**
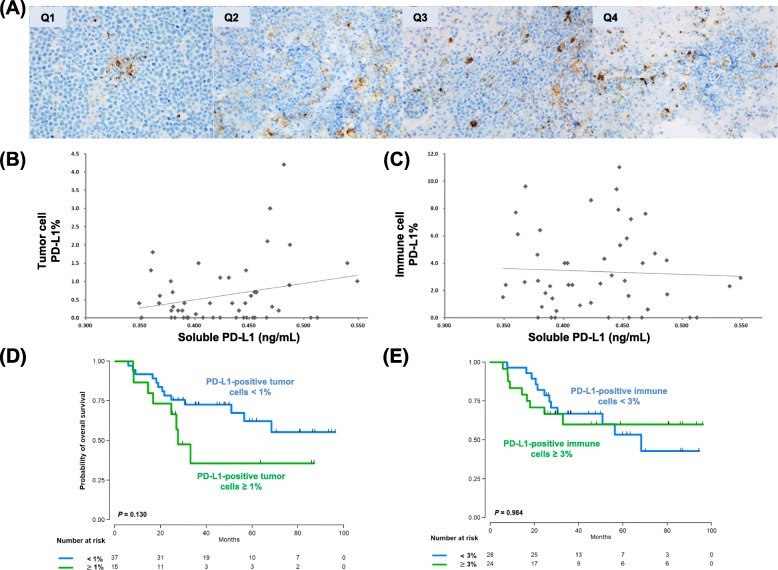
**a** The extent of PD-L1 expression in the first and second quartiles (Q1, Q2) of serum sPD-L1 was lower than that in the third and fourth quartiles (Q3, Q4). **b** The association between PD-L1-postive tumor cells and serum levels of sPD-L1 (*r* = 0.299, *P* = 0.031). **c** The percentage of PD-L1-postive immune cells did not correlate with serum levels of sPD-L1. **d**, **e** Comparison of overall survival based on the percentage of PD-L1-positive tumor cells (< 1% versus ≥1%) and the percentage of PD-L1-positive immune cells (< 3% versus ≥3%)

Out of 52 patients who were analyzed for tissue expression of PD-L1, positively stained tumor cells were found in 35 patients (67%, 35/52), whereas 17 patients did not show the presence of PD-L1-positive tumor cells. Among the 35 patients with PD-L1-positive tumor cells, the median percentage was 0.7% and 20 patients (57%) had less than 1% of positivity. The extent of PD-L1 expression in the first and second quartiles (Q1, Q2) of serum sPD-L1 was lower than that in the third and fourth quartiles (Q3, Q4; Fig. 2a). In other words, the distribution of PD-L1-postive tumor cells showed a modest association with serum levels of sPD-L1 (*r* = 0.299, *P* = 0.031, Fig. 2b). The median percentage of positively stained immune cells, including macrophages, was 2.7% (Q1: 1.2%–Q3: 6.3%); however, the percentage of PD-L1-postive immune cells did not correlate with serum sPD-L1 levels (Fig. 2c). When patients were dichotomized into high (*n* = 15) and low (*n* = 37) groups based on the percentage of PD-L1-positive tumor cells (< 1% versus ≥1%), the OS of high group did not differ significantly from that of low group (*P* = 0.130, Fig. 2e). The comparison of OS according to the percentage of PD-L1-positive immune cells (< 3% versus ≥3%), the OS did not differ, either (Fig. 2f). However, PD-L1 expression of tumor cells showed a better association with OS than that of immune cells although they were not statistically significant. In the EBV ISH using tumor tissue, the EBV-positivity was found in only one case that was positive for PD-L1. Thus, our results did not show a significant association between EBV-positivity and PD-L1 expression in patients with PCNSL.

### Serum cytokines and sPD-L1

**Fig. 3:**
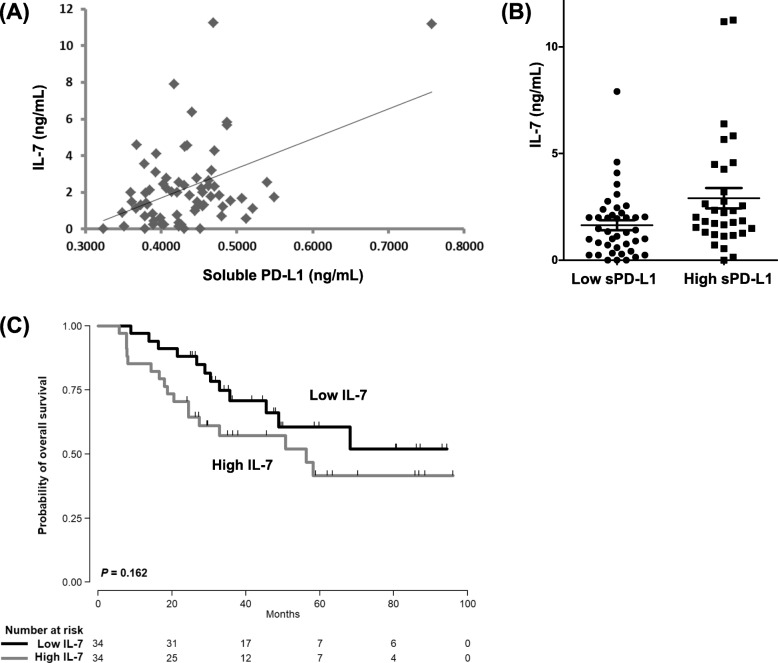
**a** The association between serum IL-7 levels and serum sPD-L1 levels (*r* = 0.521, *P* < 0.001). **b** The high sPD-L1 group showed a higher level of IL-7 than the low sPD-L1 group. **c** Comparison of overall survival based on the median value of Il-7 showed a trend of worse OS for patients in the high IL-7 group compared with those in the low IL- group

The serum levels of cytokines from 68 patients were measured to explore their association with survival outcomes and the serum level of sPD-L1. Of the 34 cytokines measured in this study, 15 cytokines (eotaxin-1, GROα, IFN-α, IL-1α, IL-7, IL-10, IL-18, IL-23, IP-10, MCP-1, MIP-1α, MIP-1β, RANTES, SDF1α, and TNF-α) could be analyzed; the remaining cytokines were not detectable in the majority of cases. In the ROC analysis for OS with these 15 cytokines, none of them showed more than 0.6 of area under the curve. Thus, an optimal cutoff for OS could not be obtained, and their levels were not associated with OS. However, the serum level of IL-7 did correlate with the serum level of sPD-L1 (*r* = 0.521, *P* < 0.001, Fig. 3a): the high sPD-L1 group had higher IL-7 levels than the low sPD-L1 group (Fig. 3b). The comparison of OS based on the median value of Il-7 showed a trend of worse OS for patients in the high IL-7 group compared with those in the low IL-7 group, but that trend was not statistically significant (Fig. 3c).

## Discussion

Since an association between sPD-L1 and prognosis was demonstrated in patients with renal cell carcinoma, the prognostic implications of sPD-L1 have been suggested in several solid cancers and hematologic malignancies [25–29]. A meta-analysis evaluating eight studies and 1102 patients with cancers of the lung, stomach, liver, and biliary tract; lymphoma; and myeloma indicated that a higher level of sPD-L1 was associated with worse OS (HR = 1.60, 95% CI: 1.21–1.99) [30]. However, the clinical relevance of sPD-L1 in patients with PCNSL has never been reported. In this study, we measured sPD-L1 from the archived serum samples of patients with PCNSL, and the median level in those patients (0.429 ng/mL, range: 0.324–0.757 ng/mL) was lower than the previously reported values in other hematologic malignancies such as DLBCL (1.84 ng/mL), extranodal NK/T-cell lymphoma (2.76 ng/mL), and multiple myeloma (4.15 ng/mL) [28, 31]. The low level of sPD-L1 in PCNSL might be associated with the peculiar characteristics of PCNSL: tumors confined to the CNS have relatively small volume, and the blood-brain barrier might influence the level of sPD-L1 circulating in blood. Nevertheless, the serum level of sPD-L1 was significantly higher in patients with PCNSL than in healthy controls, and it had prognostic value for survival outcomes. The comparison of clinical and pathological characteristics based on serum sPD-L1 levels showed no differences between the high and low sPD-L1 groups in deep region involvement, multiple lesions, cell of origin, or cell proliferation. Furthermore, even though most patients responded to the HD-MTX-containing chemotherapy, the high sPD-L1 group showed more frequent relapse or progression (78%, 25/32), resulting in more deaths (63%, 20/32, Table 2) compared with the low sPD-L1 group. These results are consistent with the relationship between poor prognosis and high expression of PD-L1 in tumor cells [32, 33]. Because overexpression of PD-L1 in tumor cells is related to the downregulation of effector T-cell function and represents a potent mechanism of tumor immune evasion [34], our findings imply a possible role for sPD-L1 in allowing tumor cells to escape from anti-tumor immunity, similar to the T-cell exhaustion through the immune checkpoint mechanism seen in the PD1/PD-L1 axis [35, 36]. Thus, patients with elevated levels of sPD-L1 might be more likely to have surviving residual tumor cells after HD-MTX-containing chemotherapy. Accordingly, the serum level of sPD-L1 could act as a reliable biomarker to predict the probability of relapse and survival outcome of patients with PCNSL.

Because circulating sPD-L1 could be secreted by PD-L1-positive cells, we analyzed the association between serum levels of sPD-L1 and the percentage of PD-L1-positive tumor cells. The distribution of PD-L1-postive tumor cells showed a modest association with serum levels of sPD-L1 (r = 0.299, *P* = 0.031, Fig. 2b) whereas the percentage of PD-L1-postive immune cells including macrophages did not correlate with serum sPD-L1 levels (Fig. 2c). In line with those findings, the survival analysis showed a trend of better OS in patients with low PD-L1 expression compared to high PD-L1 (*P* = 0.130, Fig. 2e), and the extent of PD-L1 expression in immune cells showed no difference between low and high groups (*P* = 0.984, Fig. 2f). However, these results were not statistically significant. Although there might be various causes for this discrepancy between PD-L1 expression in tumor tissue and sPD-L1 level in serum, the absence of an optimal cutoff for PD-L1 expression in PCNSL might have influenced our results. To date, no optimal cutoffs have been established, and various cutoffs for PD-L1 have been used in PCNSL patients (Table 3). This might be associated with differences in PD-L1 antibody clones, immunohistochemistry protocols, and the scoring systems used. Considering the discrepancy of serum sPD-L1 with tumor tissue PD-L1 in terms of the association with survival, further study with larger study population should be performed to evaluate whether the measurement of sPD-L1 in serum could be a feasible and reproducible test for predicting the prognosis of PCNSL as well as the status of tissue PD-L1 expression. In addition, our results were from the single-center prospective cohort analyzing relatively small number of patients although PCNSL is a rare disease entity. Thus, our findings should be validated in an independent cohort study in the future.

**Table 3:** Summary of studies evaluating PD-L1 expression in primary CNS lymphoma

	Type of specimen	PD-L1 clone	Cut-off for PD-L1 expression	Staining pattern	Frequency of PD-L1 expression
Berghoff et al [37]*.*	Whole slide	Clone 5H1 (Abcam)	≥ 5% of tumor cells	Membranous	2/20 (10%)
Four et al [38]*.*	Tissue microarray	Clone SP142 (Ventana)	≥ 1% of tumor cells	Membranous and cytoplasmic	12/32 (37.5%)
Hayano et al [39]*.*	NA	Clone E1L3N (Cell Signaling Technology)	NA	Membranous	2/64 (4.1%)
Cho et al [40]*.*	Tissue slide	Clone ab58810 (Abcam)	≥ 100 cells/HPF of tumor cells	NA	10/76 (13.2%)
Y Sugita et al [41]*.*	Tissue slide	Clone EPR1161 (Abcam)	no staining (−); 0–30% (1+); 30–60% (2+); > 60% (3+) in both tumor cells and TAM	Membranous	12/17 (70.6%) in EBV- positive cases. 11/22 (50%) in EBV-negative cases.

*TAM* tumor associated macrophages, *HPF* high power field, *NA* not available, *EBV* Epstein-Barr virus

In this study, we also analyzed the association between cytokine profiles and survival outcomes and serum sPD-L1 levels in patients with PCNSL. Cytokines in the tumor microenvironment contribute to the growth and survival of tumor cells, and we previously demonstrated an association between inflammatory cytokines and the outcomes of lymphoma patients [42–44]. However, the 34 cytokines evaluated in our study failed to show a significant association with the survival outcomes of PCNSL patients. On the other hand, serum levels of sPD-L1 were significantly related to serum IL-7, which is mainly produced by non-lymphoid cells regulating T-cell receptor γ rearrangement [45]. Our results were consistent with a previous study reporting that γ-chain cytokines such as IL-2, IL-7, IL-15, and IL-21 could induce PD1 and PD-L1 expression [46]. Thus, although IL-7 failed to show a significant association with survival outcomes in PCNSL, it might increase the serum levels of sPD-L1 by increasing the tissue expression of PD-L1.

## Conclusions

In conclusion, our study demonstrated that serum levels of sPD-L1 could reflect the expression of PD-L1 in PCNSL tumor cells and predict the survival outcomes of patients. Thus, sPD-L1 in serum could be a feasible biomarker for determining risk-adapted treatment strategies for PCNSL patients, including the use of PD-1 inhibitors. Further study with an independent cohort should be performed to validate our results in the future.

## Data Availability

All data generated or analyzed during this study are available from the corresponding author on reasonable request.

## Abbreviations

BBB: Blood-brain barrier
HD-MTX: High-dose methotrexate
IFN: Interferon
IP-10: Interferon γ-induced protein
MCP-1: Monocyte chemoattractant protein 1
MIP-1α: Macrophage inflammatory protein-1α
PCNSL: Primary central nervous system lymphoma
PD-1: Programmed death-1
PD-L1: Programmed death-1 ligand
RANTES: Regulated on activation T cell expressed and secreted
ROC: Receiver-operating characteristic
SDF1α: Stromal cell-derived factor 1α
TNF)-α: Tumor necrosis factor (TNF)-α
WBRT: Whole brain radiotherapy
